# Policymaker-led scenarios and public dialogue facilitate energy demand analysis for net-zero futures

**DOI:** 10.1038/s41560-025-01898-3

**Published:** 2025-11-20

**Authors:** Maria Sharmina, Oliver Broad, John Barrett, Christian Brand, Alice Garvey, Harry Kennard, Jonathan Norman, James Price, Steve Pye, Jack Snape, Emily White

**Affiliations:** 1 Tyndall Centre for Climate Change Research, School of Engineering, University of Manchester, Manchester, UK; 2 UCL Institute for Sustainable Resources, University College London, London, UK; 3 Sustainability Research Institute, School of Earth and Environment, University of Leeds, Leeds; UK Energy Demand Research Centre, Sussex University, Brighton, UK; 4 Environmental Change Institute, University of Oxford, Oxford, UK; 5 University of Texas at Austin, Austin, TX USA; 6 UCL Energy Institute, University College London, London, UK; 7 Government Office for Science, Salford, UK; 8 Department for Science, Innovation and Technology, London, UK

**Keywords:** Politics and international relations, Environmental social sciences, Decision making

## Abstract

Demand-side energy reductions have so far received less policy support than supply-side net-zero technologies. Here we undertake a demand-focused process for energy scenario analysis, led by policymakers and evaluated through public dialogue. We codesign, describe and model four societal futures that aim to achieve the UK’s 2050 net-zero target. The uniquely close involvement of policymakers leading the project generates markedly different narratives that reflect policymakers’ concerns while still leading to scenarios with reductions in energy demand of 18–45%—exceeding what policies normally suggest. By 2050, technology-focused systems cost 20–100% more than lower-demand ones. While intensive cocreation requires more complex interactions compared with academic-led research, it provides space for important, and otherwise absent, energy demand conversations. This work demonstrates how engaging policymakers to colead energy scenarios can challenge conventional policy assumptions on energy demand while offering an approach to support global climate mitigation efforts.

## Abstract (web summary)

New research finds that policymakers coleading energy scenario design has the potential both to better align scenarios with policymakers’ priorities and to question current policy, by substantially reducing energy use at half of the cost of technology-focused approaches.

## Main

Meeting international and national climate goals requires substantial reductions in energy demand^1,2^. Energy demand reductions of ~50% by 2050 compared with today are possible while maintaining essential services and improving quality of life^3^. Despite evidence of the benefits of such reductions, policies explicitly targeting large energy demand reductions remain scarce^4^, suggesting that they have so far been disregarded by policymakers owing to real or perceived lack of political feasibility. Instead, national energy strategies frame shifts in demand through an emphatically technological lens, focusing on efficiency gains through electrification and overlooking the broader structural and societal changes necessary to substantially cut the need to use energy^5–8^.

To address the persistent gap between academic energy demand scenarios and the scarcity of corresponding energy policy, we expand on the method developed by Barrett et al.^3^, replacing the ‘academic scenario design’ stage by a policymaker-led process with input from energy-system modellers. This process builds on the story and simulation approach^9–11^ but differs from existing participatory-scenario literature that is typically academic led and stakeholder informed. By combining the deep involvement of policymakers with a focus on energy demand, this analysis gives centre stage to incumbent views while still challenging them to think beyond typical policy priorities^12^. This key feature distinguishes our work from existing literature, describing an approach that foregrounds energy demand and societal change in a policymaker-led scenario analysis to complement existing supply-focused government studies.

The future pathway narratives that are cocreated through this process are then iteratively integrated with both sectoral and whole systems modelling, ensuring that the resulting analysis reflects policymakers’ perspectives, priorities and implicit knowledge of energy governance. To strengthen the democratic mandate for these low-carbon futures, we engage the public to deliberatively evaluate the futures^13,14^. The resulting narratives avoid being perceived by policymakers either as ideologically driven or as theoretical academic exercises. We show that demand-focused analysis conducted in close partnership with policymakers can yield markedly different and more pragmatic scenario outcomes than those developed in academia alone.

This research uses the UK as a case study, building on foundational work by Barrett et al.^3^. The UK is a country with extensive energy scenario history^15^ and pioneering energy demand research (see, for example, ref. ^16^). It offers existing case study material, active energy research network and government counterparts able to provide access to policymaker expertise and understanding of policy priorities. In this context, our policymaker-led research design can bridge the gap between academic scenario exercises and policymaking. While being UK focused, this approach has international relevance when looking to actively shape the future of global energy systems, by ensuring that academic research percolates into policy circles.

## Policymaker-led net-zero futures for the UK

**Fig. 1:**
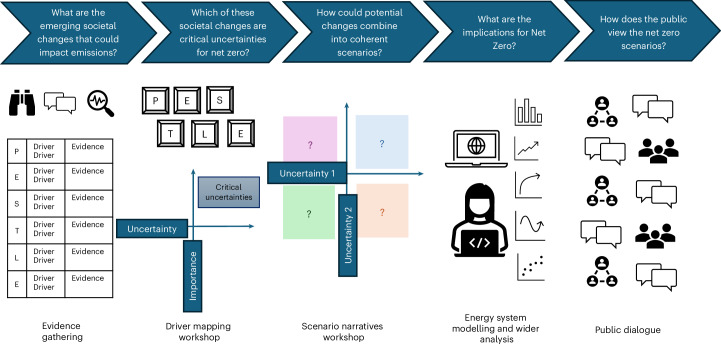
Five-step policymaker-led process for energy scenario analysis. Each of the five panels represents a step in the methodology adopted in this Article for codeveloping and modelling net-zero futures for the UK society. These steps are explained in Methods. Figure adapted from ref. ^17^ under an Open Government Licence version 1.

**Fig. 2:**
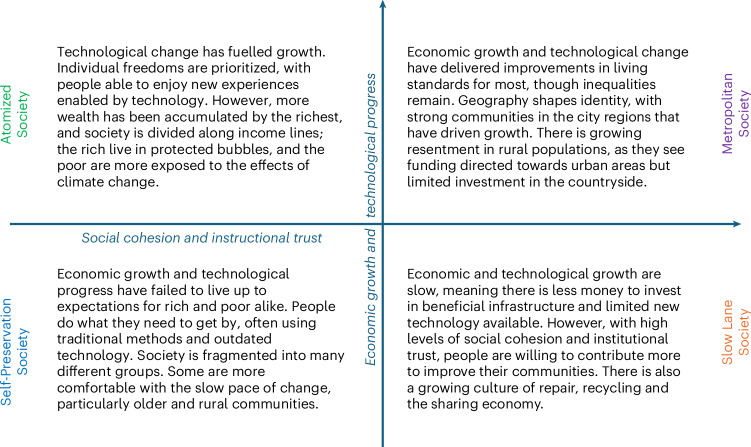
A scenario matrix of the four Net Zero Society futures for the UK in 2050. The four scenarios are distinct along the two axes of social cohesion and institutional trust, and economic growth and technological progress. Figure adapted from ref. ^17^ under an Open Government Licence version 1.

Our approach (Fig. 1) combines existing literature on societal change drivers with implicit policymaker knowledge of energy governance and their expectations for possible energy futures. We collate a longlist of drivers that could affect future demand and rank them collaboratively with policymakers and expert stakeholders (see Methods for more detail). We then group and combine them thematically along two dominant uncertainty axes, validated by expert stakeholders. The ‘social cohesion and institutional trust’ axis considers the strength of connections between individuals, institutions and business. The ‘economic growth and technological progress’ axis describes whether societies might harness economic growth, spurred by the adoption of new technologies, to their benefit. These axes help to frame the scenario matrix and narratives (Fig. 2).

Our scenario narratives describe possible future changes to UK society reflecting shared policymaker knowledge and concerns on different paths to net zero^17^. Like Barrett et al.^3^, we explore critical uncertainties in societal energy use, consumption and technology availability. However, we do not focus exclusively on demand reduction (unlike, for example, in refs. ^1–3^). Instead, these demand-centric scenarios allow wider policy considerations around emissions, growth, technology and others to play out.

**Fig. 3:**
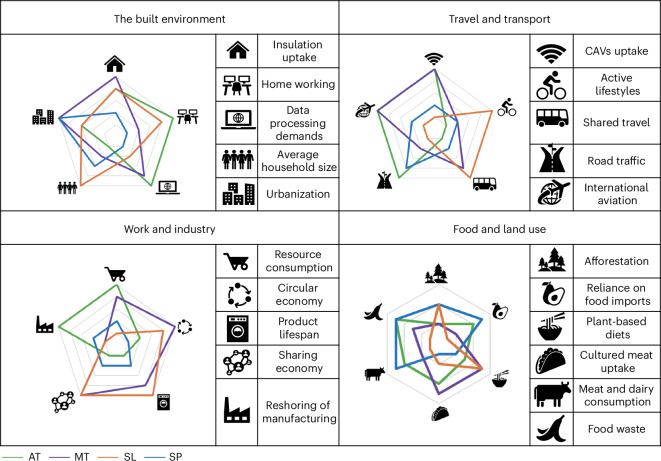
Characteristics of the four Net Zero Society futures by sector. The key sectors include the built environment, travel and transport, work and industry, and food and land use. AT, Atomized; MT, Metropolitan; SL, Slow Lane; SP, Self-Preservation; CAVs, connected and autonomous vehicles. Figure adapted from ref. ^17^ under an Open Government Licence version 1. Source data

The main characteristics of the Net Zero Society futures (Fig. 3) reflect a politically widely held view that economic growth, technological development, consumption and prosperity go together. For example, Atomized Society is digital with a shared, immersive virtual reality supported by rapid technological development. High consumption reflects individual freedom and fuels the economy, increasing quality of life but leaving some behind in the wake of automation and leading to high levels of inequality. Metropolitan Society goes further, assuming that material and energy efficiency gains allow high consumption in a future where gross domestic product, emissions and material extraction are decoupled. Trusted artificial intelligence (AI) and automation enable low-carbon lifestyles by design. Where technological development and growth are lower, societies either swap consumption for better environmental and well-being outcomes (Slow Lane Society) or face repeated recessions, unable to capitalize on opportunities that Metropolitan and Atomized Societies seize (Self-Preservation Society).

Previous research explored societal changes that could lower energy service demands and shrink the energy system without affecting quality of life^3^. Here, policymaker-led scenarios describe changes to demands as they adapt to the future state of the economy. Slow Lane prioritizes repair and maintenance over newly produced goods, but consumption falters because people cannot afford it. Lower average income has negative impacts, for example, on the public purse or means that people share transport through necessity.

As a result, the pathways are designed to be plausible but challenging. They present alternative futures considered realistic by policymakers, and modelling results are within the range of other UK analyses. However, the descriptions they use may seem unappealing, ambitious, difficult to deliver, critical or value laden. Their range of outcomes and the scenario matrix, however, were seen by policymakers as offering a useful and realistic backdrop for testing net-zero policy. The challenges these pathways raise reflect issues that any government would probably need to address in the future.

## Scenario modelling for the four UK net-zero futures

Here, we show how cocreated scenarios shape a future energy system and its ability to achieve net zero by 2050. Reviewing results from our policy-led scenarios and contrasting them with existing UK analyses, including previous low-energy-demand work^3^, highlights that designing demand-focused frameworks with policymakers can lead to markedly different outcomes. First, considering the structure of energy demand, rather than supply alone, reduces final energy needs in 2050, despite policymakers’ preference for growth. Nevertheless, scenarios are diverse and energy use trends are strongly narrative dependent, making the outcome distinct from other energy demand analyses. Second, demand-focused scenarios are not inherently low risk in terms of meeting net-zero targets. However, openly discussing drivers of energy consumption can foster conversations about the risks they reveal. Third, our scenarios have very different implications for early infrastructure decisions and corresponding potential for system lock-in or lock-out. This outcome aligns with other scenario exercises, suggesting that a policymaker-led approach does not mitigate these challenges.

**Fig. 4:**
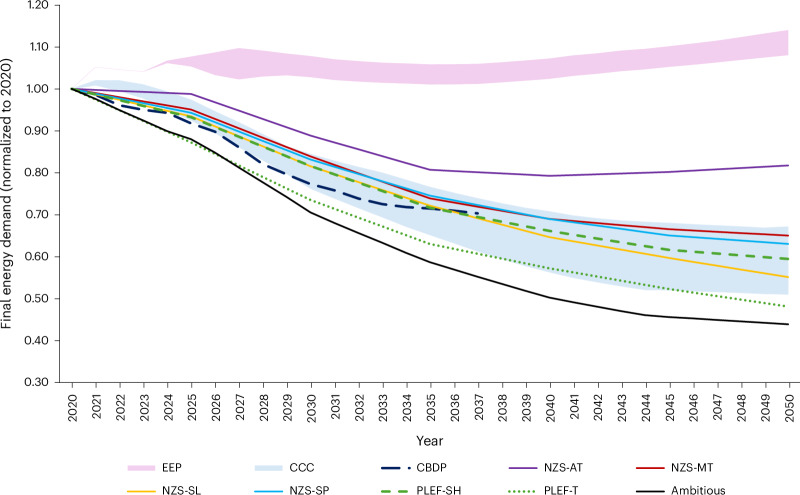
Comparison of total final UK energy demand across the four Net Zero Society futures with other published scenarios. The comparators include the ranges occupied by the CCC; government Energy and Emissions Projections (EEP) 2024, and Barrett et al.^3^; CBDP, Carbon Budget Delivery Plan; NZS-AT, Net Zero Society-Atomized; NZS-MT, Net Zero Society-Metropolitan; NZS-SL, Net Zero Society-Slow Lane; NZS-SP, Net Zero Society-Self-Preservation; PLEF-SH, Positive Low Energy Futures-Shift; PLEF-T, Positive Low Energy Futures-Transform. Figure adapted from ref. ^55^ under a Creative Commons license CC BY 4.0. Source data

Final energy consumption is systematically lower in 2050 compared with today, dropping between 18% (Atomized Society) and 45% (Slow Lane Society) (Fig. 4). Trends for Metropolitan, Self-Preservation and Slow Lane align with Climate Change Committee (CCC) Carbon Budget 6 projections, with Slow Lane closest to Barrett et al.^3^ in energy demand reduction. All our scenarios undercut government Energy and Emissions projections^18^, representative of current policy outcome, and the trajectory suggested by the Carbon Budget Delivery Plan^19^, although these stop in 2035. Nonetheless, future energy demand trends remain strongly dependent on societies’ everyday life.

Overall, these dynamics show that a demand-centric framework leads to much lower final energy consumption, even when describing markedly different narratives of which the primary focus is not demand reduction. We showed that differences in sector activity between scenarios could make a substantial difference to energy demand in 2050. The results suggest that policymaker views are not inconsistent with lower energy demand. While long-term energy needs resume growth in Atomized Society, applying policymaker views through a demand-focused approach does in most cases temper future demand expectations, compared with other government-led analyses.

**Fig. 5:**
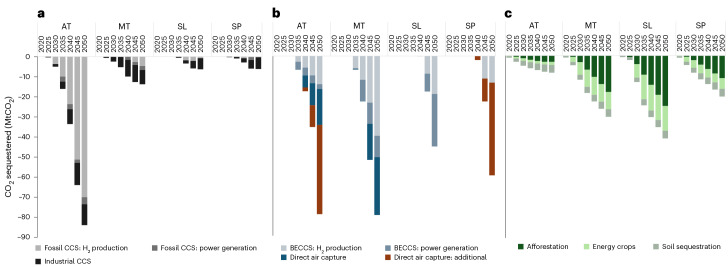
Carbon sequestration in the four Net Zero Society futures (in MtCO_2_). **a**, Carbon mitigation including CCS from fossil fuels and industrial CCS. **b**, Engineered carbon removals including BECCS and direct air capture. **c**, Nature-based carbon removals including afforestation, energy crops and soil sequestration. Source data

Net-zero emissions by 2050 remain challenging, and our scenarios all show heavy reliance on novel (engineered) and conventional (land-based) carbon dioxide removal (CDR) (Fig. 5). Novel CDR represents <0.1% of global CDR today, removing 1.3 MtCO_2_ annually with slow industrial scale-up^20^, yet it delivers between 45 MtCO_2_ per year in Slow Lane and 80 MtCO_2_ per year removal in both Atomized and Metropolitan in 2050. In Atomized Society, an additional 84 MtCO_2_ per year in mitigation (fossil carbon capture and storage (CCS)) also balances emissions in 2050. Correspondingly, combined land-use conversion rates required to support conventional CDR in energy crops and forestry range from 5 kha per year (Atomized) to 128 kha per year (Slow Lane) by 2050. For context, new forest planting in the UK has not exceeded 15 kha per year since 2004 (ref. ^21^), and areas of second-generation energy crops increased by 1.6 kha since 2018 (ref. ^22^). High growth futures (Atomized and Metropolitan) require 75% more engineered removals than Slow Lane, implicitly linking societal progress and economic growth with CDR availability. Further, 44 and 46 Mt of additional direct air capture are included in 2050 for each low-trust future, respectively (Atomized and Self-Preservation). This measure ensures all scenarios meet net zero, bridging the divide between policymaker expectations regarding whether they would and multiple scenario narrative failures that initially left this target out of reach. While high-trust futures (Metropolitan and Slow Lane) reach net zero more easily, both require substantial contributions from bioenergy with CCS (BECCS), offering 64% and 100% of their respective removals (Fig. 5).

Barrett et al.^3^ concluded that reducing energy use would reduce emissions and, hence, the need for carbon removals and suggested this approach represented lower-risk approaches to meeting our net-zero targets. While Slow Lane supports this statement, our results overall suggest that this is not an intrinsic characteristic of demand-focused analyses and that policy-centric interests may explore very different outcomes.

**Fig. 6:**
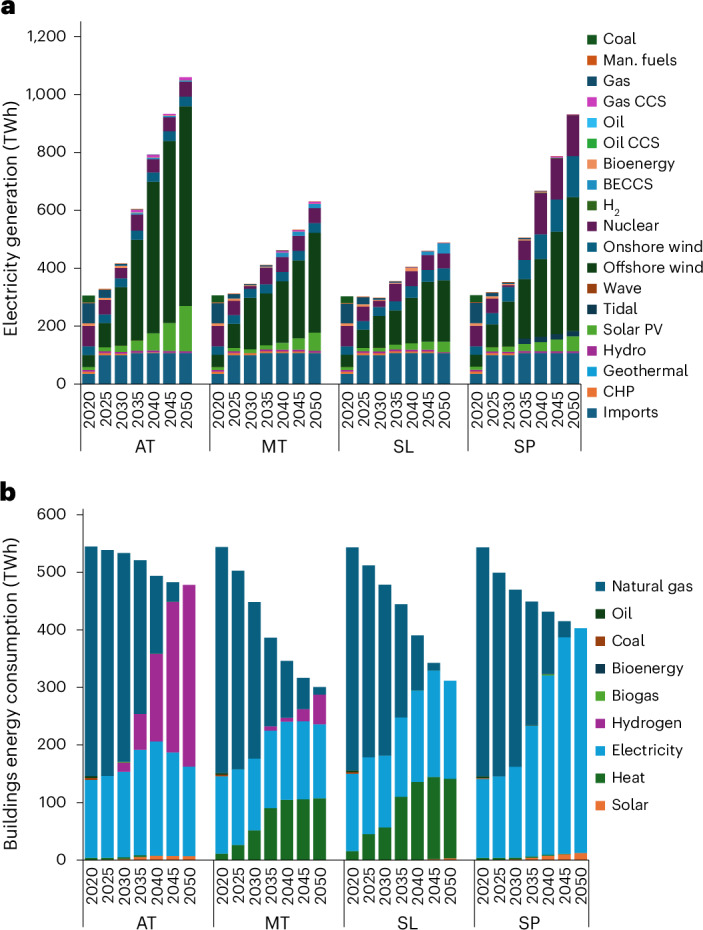
Electricity generation and residential building-sector energy consumption in the four Net Zero Society futures (in TWh). **a**, Electricity generation. **b**, Energy consumption in the residential building sector. Man. fuels, manufacturing fuels; CHP, combined heat and power; PV, photovoltaic. Source data

These differences, along with wider energy system divergence between scenarios, highlight that pathway-defining uncertainties have stark implications for large infrastructure investments and potential lock-in or lock-out. Demand for electricity in 2050 ranges from 490 TWh (Slow Lane) to 1,060 TWh (Atomized), a 216% difference mirroring additional capacity needs of 206 GW in Atomized Society (2.8 times the size of Slow Lane) (Fig. 6a).

Interestingly, power sector size is not clearly driven by growth assumptions, with Metropolitan Society being much closer to Slow Lane (30% higher electricity consumption in 2050). Instead, it responds to specific scenario characteristics: the growth of specific sectors, such as AI, with high needs for power (Atomized); or the failure of key technologies, such as CDR, pushing deep electrification to limit residual emissions (Self-Preservation).

Infrastructure decisions with potential for lock-in are also clear in the residential sector, with the competing options of communal district heat, hydrogen or electricity displayed unequally across different futures (Fig. 6b). On the one hand, widespread uptake of hydrogen in Atomized Society provides 12% of residential heat as early as 2035, rising to 66% by 2050, signalling early and sustained needs for infrastructure investment for hydrogen supply and distribution. In the other scenarios, either communal heating is widespread (Metropolitan and Slow Lane), or all heating is deeply electrified to aggressively cut emissions (Self-Preservation). These outcomes would require imminent infrastructure investment starkly different to a hydrogen-heavy future (Atomized).

**Fig. 7:**
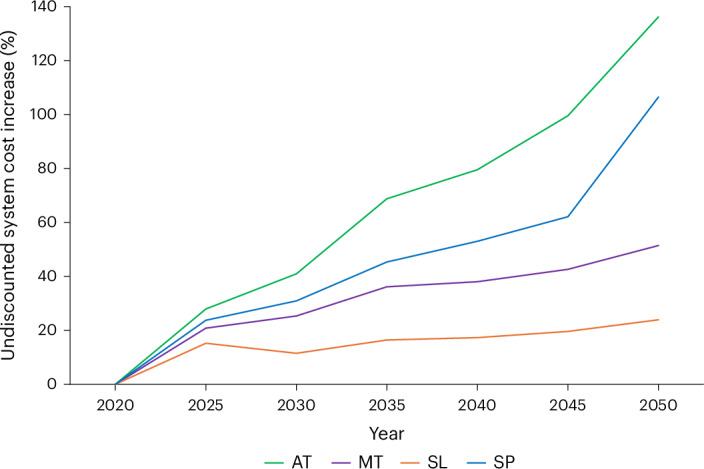
Undiscounted annual system cost in the four Net Zero Society futures relative to current levels (in %). The numbers include investment and operation costs. Source data

Variations in system size and design under each scenario reflect considerable differences in assumptions leading to very different annual costs (Fig. 7). Relative to current values, results in 2050 range from +24% to +136% in Slow Lane and Atomized Society, respectively. While the former overlaps with the +30% ‘Shift’ scenario in Barrett et al.^3^, the latter exceeds the low ambition ‘Steer’ (+68%), and neither come close to ‘Transform’ (−0.7%). In absolute terms, the annual cost of meeting net zero can be more than halved in 2050 in a lower-demand scenario (Slow Lane) compared with a high consumption, high technology and high-growth scenario (Atomized). While Metropolitan Society suggests higher growth futures are possible at lower system cost, it remains >20% more expensive than Slow Lane Society where growth is not a societal focus. Investment represents 60–70% of annual undiscounted expenditure in all scenarios. Absolute investment needs are the lowest under Slow Lane Society where the energy system is the smallest in response to lower energy needs. Overall, this range in system costs offers clear insight into the benefits of futures with reduced energy demand, contrasting them with the implications of growth-focused views.

## Public dialogue about the scenarios

The public dialogue focused on the plausibility and impacts of the four scenarios. Participants judged plausibility largely on the basis of how similar each scenario was to today’s world. For example, Metropolitan Society and Self-Preservation Society were perceived as the most likely, whereas Atomized and Slow Lane Societies were perceived as aspirational—the latter in strong contrast with its description by policymakers. The net-zero target itself was seen as less realistic in the scenarios with high energy demand arising from high material consumption, air travel and private road transport (Atomized Society). The public viewed four conditions as necessary for greater plausibility: investment, re-skilling, change in diet and change in business practices.

Impacts of the scenarios came up in four cross-cutting themes seen by participants as causes for both concern and hope: impacts of advanced technology; unequal impacts based on income and location; health impacts arising from diets and social isolation; and impacts on people’s involvement, choice and convenience. For example, the public were concerned that AI and virtual reality (Atomized Society) might lead to social isolation, although they hoped that these technologies would reduce emissions from transport.

Participants expected the government to facilitate the net-zero transition and mitigate negative impacts on incomes, health and employment. However, mistrust of government was apparent in scenarios with high levels of advanced technology and automation (Metropolitan Society), which had assumed high trust in institutions in the first place. Regardless of the scenario, the public wanted to see a consultative and place-based approach to net zero that would engage diverse views.

## Discussion and conclusion

This research uses intensive cocreation between academic and policy communities to explore demand-led scenario analysis. The futures we describe focus on possible societal pathways with intrinsically different energy needs. We show that reductions in energy demand across these futures range from 18% to 45%, strengthening the case for net-zero transition pathways to actively include demand-side measures alongside more traditional supply-side solutions. While policymaker-led analyses that focus on energy demand are rare, we show that such initiatives are possible and can produce outlooks where substantial decreases in energy demand can help to meet emission reduction targets.

Adding to previous scenario research^3,9–11^, we develop a template for academic modellers to collaborate with policymakers in a way that brings demand-side analyses up the policy agenda, increasing the impact of research and widening the role of demand-led scenarios. We demonstrate that this approach can offer outcomes that question the dominant policy focus on energy supply, while remaining more policy-relevant than purely academic work might be. Our scenarios reflect fundamentally different interests both to academic-led scenarios and to most energy policy. The framework is structured along the axes of societal trust and economic growth, mirroring traditional policy concerns linked to navigating complex trade-offs between balancing economic growth and reaching environmental goals^2,7^. At the same time, our cocreation process has allowed for the questioning of prevailing policy views on energy demand, rather than treating consumption as sacrosanct and necessary for the economy^4,23,24^. The uniquely deep involvement of policymakers in the process has shaped the key focus of the analysis, offering perspectives likely to land better with specific government departments and their existing world views.

By creating a space for cocreation and constructive challenge, our approach brings discussions around the risks and cobenefits of net-zero action closer to policy. While these risks and cobenefits have been demonstrated before (for example, refs. ^25–27^), the challenge is in translating the research into public policy. Policymakers have a key role in climate change mitigation and hold intrinsic knowledge of climate governance systems and their capabilities^28^. Recognizing this role, our approach adapts an existing demand-focused method^3^ to be policymaker-led and develops detailed qualitative narratives of possible demand-centric future pathways. Translating these into structured modelling, academics can then highlight risks that might undermine emission targets, scoping wider than policymakers may initially have expected.

We reinforce the fact that demand-side measures can help reduce societal risks by decreasing future reliance on technologies currently unproven at scale^3,25,27^, in the context of a policymaker-led framework. Our scenario matrix reflects policy priorities that favour technological progress and economic growth as a source of tax revenue^29^ and additional private investment^30^. With this framing, we show that technology-focused futures continue to see growing energy demands, display high reliance on new technology (direct air capture, synthetic aviation fuel or hydrogen) and cost between 20% and 100% more than lower-demand systems by 2050. In two of our futures, unanticipated additions of direct air capture are required to meet net zero. By contrasting these outcomes with the benefits and trade-offs that might have been expected, this work highlighted for policymakers how challenging higher-demand futures could be.

Finally, our study joins a growing body of research evidencing how public support for low-carbon activities can benefit policy design for societal change^31,32^. Our public dialogue highlights: citizen’s support for investment, re-skilling and dietary change making scenario outcomes more plausible; their scepticism that government leadership would materialize; and their challenge to the foundation of the scenario matrix itself. The inclusion of the public dialogue in a policymaker-led process reflects the importance that some policymakers attach to testing net-zero measures with the public. We suggest that any future policymaker-led demand-focused initiatives would benefit from iterative public engagement, for example, through citizen panels.

We recognize that many factors beyond those discussed here will affect policy design and decision-making on any pathway to a future that mitigates our national and global impacts on climate change. However, our research opens a constructive dialogue to answer these questions together with those who are delivering our future systems, those who research or analyse them and those who will be affected by them. Further work then should continue to bridge the gap between disciplines, recognizing the paramount place of social science when engaging different publics about net-zero policies. The value of such interdisciplinary approaches goes well beyond this UK-based case study and can inform people-centric demand-side policies internationally to enable their delivery.

## Methods

### A five-step approach to codeveloping and modelling net-zero futures

This Article builds on the multi-step low energy demand framework (LED-F) developed by Barrett et al.^3^. Here, we first describe how we replaced the first stage of the LED-F, which derived descriptions of observable societal trends with implications for energy demand, with a policymaker-led cocreation method focused on identifying and prioritizing drivers of greenhouse gas emissions as the basis for scenario storylines. We then clarify how we aligned the remaining stages of the LED-F with the outcome of our cocreation method, that is, the four scenario storylines, making key updates where relevant. Finally, we explain how we included a public dialogue, investigating public reactions to the scenario storylines that this work produced, as an additional step to the LED-F. Our approach is shown in Fig. 1, and more detailed information can be found in Bermingham et al.^17^.

A distinct element of our study compared with most academic-led research projects was an ongoing review by civil servants and by external experts, known in the policy world as ‘quality assurance’. The ongoing review included monthly submissions of drafts followed by presentations to the working group of junior civil servants, quarterly submissions of drafts followed by online presentations to a group of senior civil servants and at least 6-monthly online presentations to an external group of academic and industry experts (‘expert advisory group’ (EAG)). While a core of external experts attended all EAG meetings, the composition of the EAG varied depending on whether the scrutiny was needed for the energy modelling or for the public dialogue. For the latter in particular, the EAG included experienced social scientists independent of the project team, helping to scrutinize the ethical and methodological aspects of public dialogue study design. The EAG members were also invited to attend the public dialogue workshops as observers. All study participants took part with informed consent, although this study did not go through an ethics committee, as it was led by a government department. The EAG and quality assurance described above is the nearest government equivalent to an ethical review. At the final stage of the project, a draft project report was reviewed by civil servants from a range of government departments to ensure consistency and relevance to policies outside the emissions and energy remit.

### Steps 1–3 cocreating scenario storylines

Our methodology included five steps (Fig. 1). In step 1, we carried out a desk-based evidence-gathering exercise to understand how societal changes can impact energy use and greenhouse gas emissions. This step included horizon scanning and a rapid literature review to answer the question, ‘What are the main drivers of societal and behavioural change that will directly or indirectly affect UK greenhouse gas emissions between now and 2050?’. We used peer-reviewed journal articles, grey literature and the news media to shortlist 40 drivers of emissions. Unlike the LED-F work that prioritized drivers of energy demand^3^, our focus was primarily on emissions in line with the UK’s net-zero emission target. We then categorized the drivers of greenhouse gas emissions as political, economic, societal, technological, legislative and environmental using a PESTLE (Political, Economic, Social, Technological, Legal and Environmental) framework^33^. Examples of drivers prioritized by expert stakeholders at the next step of our methodology to feature most prominently in our scenarios included relative costs of making the low-carbon choice, localization of production and economic activity and the use of connected and autonomous vehicles. The full list of drivers is provided in Supplementary Table 1.

Step 2 was to prioritize the most important and the most uncertain drivers of greenhouse gas emissions. To this end, a two-part facilitated online workshop was held across 2 days in February 2022, each part being 2.5 h long, which brought together 35 expert stakeholders with relevant expertise from national government, local government, industry, third sector organizations, citizen groups and academia. We recruited participants through email-based snowballing reliant on existing networks both internally to government departments and with external (academic, industry and third sector) contacts. The participants were selected by the project team as a sample of convenience, primarily on the basis of their expertise in low-carbon societal transitions and their availability to attend the online workshops. This sampling technique was chosen owing to a relatively low number of experts (elite participants) and their limited availability to take part in the study.

Workshop participants were divided into six virtual breakout groups of four to five people each. Participants’ contributions were recorded on a Mural board, in addition to notes taken by the project team in each virtual breakout room. During the first part of the workshop, participants scored the importance and uncertainty of each driver on a scale from ‘unimportant’ to ‘highly important’ and from those with very high uncertainty to very high certainty in the direction of their future development. The scores were then combined with qualitative feedback from the participants to identify which drivers were both highly important and highly uncertain and would therefore be considered as critical uncertainties. A full list of the critical uncertainties is provided in Supplementary Table 2.

During the second part of the workshop, participants developed the critical uncertainties into 18 ‘axes of uncertainty’, which explored two alternative outcomes for each critical uncertainty that were both plausible and divergent from each other. Following the workshop, we identified common themes and relationships within the 18 axes of uncertainty, converging on two dominant axes. These were ‘trust’ and ‘growth’, or more precisely: social cohesion and institutional trust, and economic growth and technological progress.

As part of step 3, a second 3-h facilitated online workshop was held in March 2022 with the same group of participants, to begin developing narratives for the scenarios. To validate the scenario axes, participants were provided with advance descriptions of each axis and of how information from the first workshop led to its selection. These descriptions included how the two axes combined to produce four future scenarios but were not so detailed as to constrain creativity.

Finally, more detail was built into the narratives to enhance their plausibility and coherence. Narratives were drafted by the project team, describing how each 2050 scenario looked and what the implications could be for people living in the UK. Expert stakeholders were consulted to ensure that the scenarios appropriately reflected the workshop participants’ views and tackled the issues expected.

### Step 4 modelling scenario storylines

The LED-F framework^3^ that underpins our work includes three integrated and iterative modelling stages that provide internally consistent interpretations of each of the scenario storylines it is applied to. These stages involve (1) individual sector-level modelling, (2) identifying relevant inter-linkages between end-use sectors and (3) integrating end-use sector analyses into a central modelling system. This section summarizes the LED-F approach and explains differences to its first use. Full details of the conceptual approach and how it was carried out in the context of modelling a family of demand-centric scenarios are available in the study by Barrett et al.^3^.

Our end-use sectors included mobility, shelter, non-domestic buildings, materials and products, and nutrition. These sectors mirrored important areas for energy use across the UK economy. As the ways in which energy demand and emissions are generated in each sector are intrinsically different, we used different dedicated models. These included TEAM-UK (Transport Energy Air pollution Model for the UK), UK National Housing Model (NHM), UK Building Energy Efficiency Survey (BEES) dataset and UK Multi-Regional Input Output (UK-MRIO) model. TEAM-UK is a simulation model of transport–energy–environment systems^34,35^ that projects future travel behaviour and vehicle use on the basis of relationships between social and technical drivers and transport demand. The UK NHM^36^ relies on National English Housing survey data to run building-physics-based simulations of how changes to current and future housing stock affect domestic energy needs. The UK BEES dataset^37^ underpins a purpose-built simulation analysis of future energy needs in the non-domestic building sector. Hybrid use of the UK-MRIO model and physical modelling investigated the impacts of changes in the nutrition^38^ and the materials and products^39^ sectors, with the former focusing on physical input–output food system modelling and the latter on supply chain and production impacts of structural changes.

Implicit dependencies between end-use sectors were accounted for to ensure internal consistency across sectoral model outputs. These reflected how scenario outcomes in one sector could have direct yet hidden implications for another. One example would be how changes in car use through shifts to public transport or active mobility affect the need for both road maintenance and new road building, thus implying changes in output from the materials and products sector. These links were codified as part of the LED-F framework and remained unchanged in this study.

Finally, the outputs from sectoral simulation models were integrated into the UK TIMES whole energy system model of the UK. UK TIMES is a technology explicit, linear optimization model that has been both used extensively by academia (for example, refs. ^40,41^) and codeveloped with government to support their statutory energy system analysis duties (for example, refs. ^19,42^). This integration ensured that long-term system-wide implications of individual sector changes, as well as the possible energy and emissions trade-offs between these sectors, were accounted for in a fully internally consistent analysis framework. At this stage, system level constraints (for example, national carbon budgets and net-zero emissions targets) and total resource use (domestic and international) were squared off and any shortfalls or discrepancies were addressed before iterating back with required updates to the sectoral models.

Three additional scenario narrative threads that resulted from the policymaker-led process were not part of the LED-F. They required updates to the modelling framework as follows. First, an option of cultured equivalents to red meat (that is, laboratory-grown meat) was introduced to the nutrition analysis. Levels of uptake varied across scenarios and were introduced as percentage changes in consumption assumed to displace conventional livestock production, shifting needs for energy, land use and emissions accordingly. Second, non-domestic buildings modelling was expanded to include projections of energy demand from growing use of datacentres. This addition reflected differing scenario views of how connected future societies are and what this connectedness implies for future energy use in this sector. Quantitative translations of each growth narrative were built by combining national energy statistics for computing electricity needs^43,44^ and National Grid Future Energy Scenario analysis for possible sector development pathways^45^. Third, the uptake of connected and autonomous vehicles was assumed to be high in digitally enabled scenarios but with different implications for equity, whereby either the rich dominated such use with much private car ownership or alternatively such vehicles were widely available, shared and often publicly owned.

### Step 5 public dialogue

The public dialogue was conducted by Ipsos on behalf of the Net Zero Society project team. It was designed to explore public views on the four Net Zero Society scenarios, including their comparative challenges, advantages and plausibility. The involvement of the public increased the policymakers’ confidence in the scenarios. In a way, the public dialogue acted as a sense check for unintended consequences. Full details about the public dialogue methodology and findings are available in ref. ^17^.

Our approach to public dialogue was informed by social science (for example, ref. ^14^), involving a sample broadly representative of the UK society, using a set of personas to evaluate unequal impacts on different members of society and treating people as citizens rather than as consumers. Ipsos recruited participants through specialist recruitment agency partners: Criteria UK (an approved Ipsos supplier and regularly used for large deliberative projects across the UK) and Field Mouse (a specialist rural recruitment agency), using on-street, telephone and online approaches, as well as snowballing.

The public dialogue deliberately included underrepresented communities, such as ethnic minorities, those with English as an additional language and those with a lower income. To ensure such inclusion, given our small sample (*n* = 30), we used purposive sampling and minimum quotas for each demographic. The demographics included gender, age, household income, location (urban or rural), housing type (owned or rented), degree of concern about climate change, attitude to technology adoption (for example, early adopters) and attitude to government intervention. Purposive sampling is often deemed to limit the generalizability of the findings, as it can be difficult to ensure that the sample is representative. In our case, however, purposive sampling was specifically used with target quotes to make the sample representative of the UK population.

The participants were encouraged to imagine themselves inhabiting the four 2050 futures, facilitated by a range of printed materials and artefacts: rich picture illustrations showing life in 2050, ‘future artefacts’ sent to participants by post in advance representing cultural and daily life elements and persona cards representing different demographic groups, particularly focusing on underrepresented perspectives.

The data collection methodology included an introductory webinar, four 3-h facilitated workshops covering each scenario separately and a final 3-h facilitated workshop bringing the scenarios together for comparison. Each participant was paid £40 for the webinar and £60 per workshop. The introductory webinar introduced core concepts including climate change, net-zero targets and emission reductions. The four scenario workshops featured breakout rooms where participants imagined themselves in the scenario, exploring different aspects including built environment, food and land use, work and industry, and transport. The composition of the breakout groups and topic sequences were varied to ensure diverse discussions. The process concluded with a final cross-scenario workshop where participants reflected on all four scenarios. This session examined graphics showing energy infrastructure implications, costs and external risk factors, while discussing scenario plausibility, tensions and potential societal changes leading to each scenario. All workshops took place online.

During the sessions, trained notetakers transcribed the participants’ contributions, with transcripts then thematically coded using NVivo software. The coding process used terms such as ‘few’, ‘some’ and ‘many’ to indicate the frequency of specific codes in the transcripts. The analysis focused on perceptions rather than facts and noted where views applied to participant subgroups with more extreme interpretations of the scenarios. The methodology acknowledged that while the four scenarios were designed to be divergent and stretching, participants often made assumptions beyond the presented information.

### Limitations

An integrated analysis of the nature presented here, bringing policy and academic environments together with blended analysis frameworks, inherently carries limitations. First, expert and stakeholder bias may arise from intensive and continuous knowledge exchange between policymakers, academics and advisory group experts, both inside and outside the project team. Our aim in engaging stakeholders was to improve the quality of decisions rather than to make scientific judgements, helping to mitigate concerns that the primacy of science would be compromised by involving non-scientists^46^. In addition, evidence shows that stakeholder engagement in research results in better problem-solving and decisions^47^. Scenario planning in particular can mitigate cognitive biases in decision-making, especially where scenarios are developed by expert stakeholders themselves rather than being presented to them^48^. To improve the quality of expert elicitation, we followed a number of strategies: inclusion of a range of expert views^49,50^, transparency of the process^50^, engaging experts in a structured way^49,51^, creating multiple scenario visions^50^ and varying the composition of breakout discussion groups^52^.

Second, our study builds on the method described in detail in the study by Barrett et al.^3^, enclosing their modelling framework within a policymaker-led approach and a public dialogue. In this way, we test how purely academic findings on potential for energy demand reduction fare under different assumptions and demand-centric scenario frameworks. We also test whether such a method is a useful way of introducing energy demand into policymakers’ frames of reference. Ensuring consistency with Barrett et al.^3^, however, implies that modelling tools and methods remain comparable between studies. So, while criticisms of linear-optimization-centric approaches abound^53^ and the use of more innovative approaches, such as agent-based modelling, may be better suited to analysing socio-technical energy transitions^54^, these fell beyond the scope of this study. Each individual model is described in the ‘Step 4 modelling scenario storylines’ section, and their limitations are addressed in the paper by Barrett et al.^3^ as well as in their foundational papers, also listed in the ‘Step 4 modelling scenario storylines’ section. Key limitations of the linking methodology relate to levels of aggregation required to pass information from sectoral to whole energy system modelling frameworks. Sectoral simulation models carry richer pictures of the changes implied by policymaker views than can be passed through to UK TIMES. For example, the food model produces rich detail on diet composition, demands for different food groups and diet uptake. However, connections to UK TIMES are limited to scenario greenhouse gas emissions, energy use in agriculture and industry, and waste projections. Similarly, the MRIO model used to represent supply chain impacts of changes to final and intermediate consumption covers 106 sectors across multiple regions. The UK TIMES model represents industry aggregated into six key subsectors for the UK. Soft-linking the two inevitably means that the diversity and relative depth of impacts within one sector in the MRIO model will be partly lost when translated into UK TIMES. These limitations are particularly important in the context of representing policymaker views, as they may either disproportionately increase or reduce the impact of specific statements, affecting how well the final analysis reflects policy-relevant scenarios. The deep involvement of policymakers in cocreating this analysis, the iterative nature of the modelling and our public dialogue all help to mitigate this outcome. Notwithstanding, future iterations of this methodology should seek to develop UK TIMES and sectoral modelling tools so as to improve soft-linking capabilities.

Third, our modelling framework, and hence our study, focuses on the UK. The design of the modelling framework will, in part, have been dictated by contextual factors including data availability, existing energy research networks and established modelling frameworks on sectoral and whole system levels. Transferring the approach to a different country may therefore imply making adjustments that account for each country’s local contexts along similar lines. However, while political systems, historical developments, societal structures and existing analysis might differ, we argue that our approach has universal themes. Most importantly, (1) the role of policymakers in shaping the governance and physical infrastructures that drive energy demand and (2) the global reluctance among policy groups to discuss demand-side measures and demand reduction, taken together, make this work relevant for other countries in their effort to meet their respective climate targets, highlighting implications for our global climate mitigation efforts.

Finally, deep cocreation with policymakers implies following governmental practices, such as retaining limited access to some of the data generated during the project. Specifically, detailed data (for example transcripts or recordings) from the expert workshops and the public dialogue remains with the government. While attributing contributions to specific participants, or sharing full transcripts of scenario codesign meetings, may often be inappropriate, releasing aggregated, anonymized or otherwise summarized versions of such data where possible would support better open-research practices. We would recommend that future iterations of this approach consider this openness where possible.

### Reporting summary

Further information on research design is available in the Nature Portfolio Reporting Summary linked to this article.

## Supplementary information

Supplementary Information Supplementary Tables 1 and 2.

Reporting Summary

## Source data

Source Data Fig. 3 Statistical source data.

Source Data Fig. 4 Statistical source data.

Source Data Fig. 5 Statistical source data.

Source Data Fig. 6 Statistical source data.

Source Data Fig. 7 Statistical source data.

## Data Availability

All results data underpinning the figures presented in this article are available in the source data. Further graphs and analysis can be found at https://public.tableau.com/app/profile/stevepye/vizzes. Further details on the data and assumptions that underpin the analysis are a matter of public record and are available from the Government Office for Science website at https://www.gov.uk/government/publications/net-zero-society-scenarios-and-pathways--2. These include: evidence reviews of Recent Societal Trends (Annex 1) and Societal Change (Annex 2) used to inform scenario design; Drivers of Change and Axes of Uncertainty underpinning the scenario matrix (Annex 3, also available in the source data); detailed Modelling Inputs across the modelling design (Annex 4); and demographic characteristics of public dialogue participants, including their numbers by gender, age, income and geographic location (Annex 6). Source data are provided with this paper.
