# Supplementary material for: Policymaker-led scenarios and public dialogue facilitate energy demand analysis for net-zero futures
Source: Nat Energy. 2025 Nov 20;10(12):1482–92. doi: 10.1038/s41560-025-01898-3 (PMC12722179; doi:10.1038/s41560-025-01898-3)
Supplement: Supplementary file 1 — Supplementary Tables 1 and 2. [file 41560_2025_1898_MOESM1_ESM.pdf]

nature energy

Supplementary information https://doi.org/10.1038/s41560-025-01898-3

Policymaker-led scenarios and public dialogue facilitate energy demand analysis for net-zero futures

In the format provided by the authors and unedited

Supplementary Table 1. The main drivers of societal and behavioural change that will directly or indirectly affect UK greenhouse gas (GHG) emissions between now and 2050

Category	ID	Driver	Description
**Political drivers**	**P1**	Political focus on climate and emissions	Climate change issues may grow in importance for voters because of increased public understanding of these issues, through coverage both in the media and school curriculum. This could indirectly lead politicians and businesses to respond with net zero policies and new products or services but might not necessarily translate directly into people making more sustainable choices because of the value-action gap.
	**P2**	Increase in local emission reduction targets and pro- climate initiatives	Local areas may increase commitments to reduce emissions ahead of the UK-wide net zero target, partly incentivised by grassroots campaigns. This might lead to some additional sustainable choices at a local level, especially if the trend towards levelling up and empowering local governments continues. Changes at a local level could also create more opportunities for central government's delivery of net zero policies.
	**P3**	Reconfiguration of democracy driven by digital participation	More inclusive and widespread participation in democratic processes could be driven by access to new technologies and public deliberation (for example, through citizen assemblies). This could provide opportunities for the public to engage further with net zero issues at a local and national level.
	**P4**	Business influence on political decision making	Reaching net zero requires change from the population, private sector, and public sector organisations. However, the extent of corporate involvement in these processes is unclear and could potentially influence political decision making. Understanding the overall direction of this as either an enabler or barrier to meeting net zero is vital in the future.
	**P5**	Polarising trust in government and institutions	In 2022, the ONS found 35% of the population stated that they trust the national government, although different levels are reported in various services provided by government. When people do not trust that decisions are being made with their interests at heart, they are less likely to be accepting of policy change. Therefore, polarised trust in the government, particularly across

			different sections of society, creates uncertainty about the extent that change can happen as a result of net zero policies.
	**P6**	Increasingly controversial and fractious geopolitics	The UK's political alliances are increasingly important in the context of ongoing conflicts, volatile energy prices and the impacts of climate change across the world. This conflict drives uncertainty in how effective multilateralism can be and could impact the implementation of global decarbonisation agreements (positively or negatively) depending on the fluctuating geopolitical state of play.
	**P7**	Proliferation of more diverse political and leadership voices on climate	More diverse voices in the public debate on climate change could lead to more 'cut through' with groups currently less engaged on the issue. This could include more under-represented groups in leadership positions within government, businesses, and other organisations (some of which may be more ambitious on climate action). This could also be countered by new, more outspoken voices on climate denial. The impact on emissions will depend on which voices resonate with more people.
	**P8**	Expanding suffrage	There continues to be debate on lowering the voting age, with several jurisdictions already having voting age of 16. Young people are more willing than adults to take action on climate change9 and may push more strongly on intergenerational justice and climate action.
**Economic drivers**	**Ec1**	Macro-economic stability and growth	A growing economy can drive production and consumption of more goods and services, and growing household incomes are associated with increased levels of travel. Although the exact impact on energy demand and emissions will depend on how quickly energy and emissions intensities are falling, more growth will mean more energy demand, all else equal. On the other hand, economic growth is also associated with investment and technological innovation, both of which are needed to meet net zero. The OBR has tended to forecast lower long-term

			growth since the 2008 financial crisis, which has been ‘baked into’ net zero pathways. The tension between this assumption and a desire to increase economic growth results in uncertain implications for emissions into the future.
	**Ec2**	Relative costs of 'making the green choice'	The ‘green gap’ describes the gap between consumers’ stated intention to act sustainably and their actual behaviours, often due to the perceived or actual cost of making a sustainable choice. A variety of factors influence this, such as rising costs, inflation, and ease of access/use. It is unclear how changes in these factors will play out over the long term and impact societal trends in sustainable (‘green’) choices. This driver is focused on factors outside of government climate policy.
	**Ec3**	Participation in the circular economy and 'as a service' economy	Circular economy business models could grow in prominence, such as consuming fewer virgin materials and keeping goods in use for longer. This could build on current right-to-repair movements and concerns about waste by households and businesses. Whilst governments will play a role in supporting circular economy trends, it is possible that they could be driven by new business practices and consumer choices.
	**Ec4**	Awareness of carbon impact of products and services	Monitoring, reporting and verification of emissions (for example through carbon foot-printing of activities) could increase and there could be growing public awareness of environmental issues.
	**Ec5**	Businesses respond to changing sentiments on climate change	Businesses' social and environmental reputation may grow in importance, increasing investment in low-carbon products and services by a range of organisations (including pension funds). Increased availability of finance could facilitate the development of new technologies or services in the environmental sector.

	**Ec6**	Potential for increased localisation of production and economic activity	Driven by supply chain disruptions during the Covid-19 pandemic, it has been estimated that the manufacture of £4.2 billion worth of products could be reshored by UK retailers between 2020 and 2021. A UCL analysis highlighted a range of factors that would determine the impact of this on emissions, on both the supply and demand side.18 For example, UK domestic energy use and emissions would likely be higher in a scenario with increased reshoring relative to a scenario with lower levels of reshoring, but this could potentially lead to the UK's overall carbon footprint being lower due to the use of less carbon- intensive energy in the UK. There could also be trends towards localisation of economic activity within the UK, driven by technology and societal trends, and triggered by such shifts during the pandemic.
	**Ec7**	Economy measured by alternatives to GDP	Indicators of a nation's progress could encompass more than gross domestic product (GDP) in the future. For example, measures of life satisfaction, anxiety ratings, and other indicators of personal and economic well-being may become more widely used. This could incentivise governments and businesses to shift the focus away from economic growth to sustainability- focused activities including circular economy actions.
**Societal drivers**	**S1**	Shifts in where and how people live – places and housing	The ONS has found that family and household structures have been constantly changing. For example, there has been an increase in single-person households and multi-family households. These changes, along with how people work (see below driver), can have a range of impacts on emissions, complicated by the diverse range in the types of housing they inhabit, which influences energy efficiency.
	**S2**	Changing nature of work (Including remote working and changing sector mix)	Advances in automation and remote communication technology, as well as changes spurred on by the COVID-19 pandemic, have changed the nature of how and where people work. The changing cost of energy may also impact whether people choose to work at home or in the office. Ongoing uncertainty in these trends creates uncertainty in energy

			use and emissions in related sectors, such as buildings and transport.
	**S3**	Health impacts linked to climate change	Threats from climate change to health could rise due to increases in flooding, longer and hotter heatwaves, and more extreme weather events. Climate- related threats to health could increase public support for net zero policies.
	**S4**	Demographic shifts, globally and within the UK	The demographic composition of the UK population is likely to change, with a growing number of over-65s and over-75s, a declining fertility rate, and lower mortality rate. Combined with potentially higher numbers of climate refugees in future, the impact of the demographic changes on emissions is uncertain.
	**S5**	Real and perceived unfairness of impacts of climate change and paying for net zero	There is a growing emphasis on how to mitigate the unequal impacts of climate change. If net zero policies disproportionately affect the less well-off and consumption increases for the richest segments of society, this might lead to a backlash against such policies. At the same time, groups experiencing the worst effects of climate change might put pressure on governments to accelerate emission reductions.
	**S6**	Increasing 'green choices' where there are health and economic co-benefits (cycling, diet, food waste)	There may be greater emphasis on the advantages of low-carbon choices beyond their impact on climate change in the future. Examples include improved health because of dietary changes and active travel, and lower material use and loss because of reductions in food waste and other types of waste. This could lead to people making sustainable choices, even if they do not prioritise sustainability as an issue.
	**S7**	Changing leisure patterns	Leisure patterns may change, and people may choose to spend more leisure time in the UK, as was seen during the COVID-19 pandemic. This would reduce demand for aviation and would shorten the average length of trips, thereby reducing emissions.

**Technological drivers**	**T1**	Potential for net zero technology cost or performance changes that move the goal posts	If low carbon technologies are adopted more rapidly than expected, such as due to lower costs and performance improvements, emissions could drastically reduce. Other technologies, such as geoengineering or direct air capture solutions, could also change government priorities in tackling climate change. However, it is uncertain (both on the demand and supply side) the extent to which these changes will occur, and whether they will be balanced out by other factors both domestic and internationally.
	**T2**	Increasing deployment of smart internet of things (IoT) devices	Increased deployment of smart meters and internet of things devices increases consumers' awareness of their energy use patterns, encourages off-peak energy use, and reduces reliance on fossil fuelled power stations at peak periods. However, the extent of behaviour change from increased awareness is uncertain.
	**T3**	Increasing use of connected and autonomous vehicles (CAVs)	Experts predict that new automobiles will have autonomous capabilities under most conditions within 10-20 years. Automation facilitates the adoption of energy-saving driving practices and changes in vehicle design that enable emissions reductions. However, heavy uptake of CAVs could increase demand for travel by car, including by non-drivers. There is also uncertainty over their impacts on reconfiguration of streetscapes and the effect this could have on public transport and active travel options.
	**T4**	Increasing use of artificial intelligence (AI) in energy systems and across the economy	Increasing deployment of artificial intelligence (AI) in electric power systems could optimise power grids and increase energy efficiency, thereby reducing emissions. However, powering and training AI in the first place is energy intensive, and there is considerable societal scepticism of AI,35 raising the question of how this tension will be balanced to optimise energy efficiency with public trust.
	**T5**	Sustained low awareness of, and consumer barriers to, renewable heating	Awareness amongst the public about renewable heating has remained below 50% between 2015 and 2021. People are put off adopting low carbon heating systems primarily due to upfront costs and uncertainty about performance.

	**T6**	Advancements in communications technologies and the metaverse	Recent advancements in communications technologies have enabled the rapid increase in remote and hybrid working required due to the COVID-19 pandemic. Continued digitalisation of work, leisure and consumption reduces emissions from transport and production, but data centre processing, cloud services, and the high-resolution imagery required by the ‘metaverse' are energy intensive.
	**T7**	Increasing data infrastructure and use, leading to new energy demands	Blockchain, in combination with smart meters and internet of things devices, enables highly accurate, traceable, and transparent measurement of carbon footprints that can be transformed in to tokens or carbon credits. However, this can also be linked to increased emissions.
**Legislative drivers**	**L1**	Increasing international commitments on emission reductions and translation into national legislative commitments	Increasing occurrence of extreme weather events such as heatwaves, severe storms, and flooding could have impacts on health, mortality, and food security. Increasing first-hand experience of the impacts of climate change could motivate individuals to demand stronger action on climate issues.
	**L2**	Potential cases of climate change litigation	The cumulative number of climate change-related litigation cases has more than doubled from 2015 to 2022. Strategic litigation that targets governments, businesses and financial actors is on the rise. Increasingly, litigation is used as a tool to encourage a 'just transition', but also as a way for fossil fuel companies to litigate against governments. Depending on the source, volume and value of these litigations, the impact on government actions and societal emissions could be substantial.
	**L3**	Financial costs for emissions	Civil penalties issued to businesses by the Environment Agency for non-compliance with climate change legislation increased from £1.4 million to £2.1 million between 2018 and 2021. UK carbon credits have been increasing since the UK Emissions Trading Scheme (ETS) started and are retailing at a higher cost than those in the EU ETS. All else equal, this might increase the financial incentive for companies to reduce their emissions, but it is not

			known to what extent this would happen, or if they could turn to offshore emission increases instead.
	**L4**	Changes to the global carbon accounting scheme	As of 2019, the UK is the biggest net importer of carbon dioxide emissions per capita in the G7. Were the global accounting regime to change significantly it could lead to changes in how national policies have to respond to different sources of emissions.
	**L5**	Increasing recognition and regulation of 'how climate will be impacted' into all legislation	Integrating an assessment of climate impacts into all legislation could lead to many small reductions in emissions that are not currently assumed in government projections.
	**L6**	Increasing co-benefits for net zero from other environmental policies	Clean Air Zones and similar initiatives disincentivise car use and encourage modal shifts to lower carbon transport options. Similarly, rewilding motivated by biodiversity concerns has co-benefits in the form of carbon sequestration by trees.
**Environmental drivers**	**En1**	Increasing awareness and experiences of extreme weather events and their impacts	Increasing occurrence of extreme weather events such as heatwaves, severe storms, and flooding could have impacts on health, mortality, and food security. Increasing first-hand experience of the impacts of climate change could motivate individuals to demand stronger action on climate issues.
	**En2**	Development and greening of liveable cities	A trend towards green and liveable cities is being seen in an effort to facilitate climate-friendly urban areas. Increasing urban tree cover, for example, improves resilience to climate change and improves perceived aesthetics and liveability of neighbourhoods. Improvements in city infrastructure also encourage active travel and discourage car use. However, it remains to be seen how widespread these developments will be in the UK and how significant an impact they will have on the behaviour of city residents.

	**En3**	Influence of environmental concerns / extreme weather on property values and internal migration	Economic theory suggests that climate-related risks (such as flooding and rising sea levels) should decrease property values in at-risk areas but, the relationship has been found to vary depending on the frequency and severity of extreme events. How this relationship develops with the increasing trends in extreme weather events and the fluctuating property market is uncertain.
	**En4**	Increasing effects of development and climate change on biodiversity	UK biodiversity could be increasingly threatened by unsustainable agricultural and woodland management, development and urbanisation, pollution, hydrological change, and invasive non-native species. Invasive species cost the economy £1.8 billion per year and are likely to increase with climate change. Efforts to improve and restore biodiversity can have climate co-benefits and encourage environmental awareness.
	**En5**	Tension in how land is used - housing, farming, power generation, afforestation, etc.	There is likely to be increasing competition between renewable energy generation, development, and urbanisation due to population expansion, afforestation, peatland restoration, and growing crops for bioenergy. However, there is uncertainty over how this will play out and how land will ultimately be used, particularly where uses such as solar, onshore wind and housing face opposition from the public being affected.
	**En6**	Disruption to food and water supply from soil degradation, warming temperatures, etc	Loss of soil carbon costs the UK £3.21 billion annually, and the productivity of soil is declining globally because of intensive farming, over-grazing, and climate change. Soil scientists estimate that global soils can only support another 60 harvests, threatening food security globally and in the UK. Disruption to food and water supply threatens political stability, livelihoods, health and wellbeing, and shifts people's priorities.

Note: For references, please see Annex 3 to Bermingham, R., Snape, J., Wells, T., Ballard, H., Blackbourn, H., Grassmann, N., Nicol, C., Sharmina, M., Taylor, M., & White, E. (2023). Net zero society: scenarios and pathways: How could societal changes affect the path to net zero? Government Office for Science. Available at: https://www.gov.uk/government/publications/net-zero-society-scenarios-and-pathways--2

Supplementary Table 2. 'Axes of uncertainty' formed of two alternative outcomes for each critical uncertainty that could directly or indirectly affect UK greenhouse gas (GHG) emissions between now and 2050

Businesses agree and act towards strict emissions targets (individual & sectoral)	⟵⟶	Businesses act against targets and regulation
Increased number of legal losses for the environment	⟵⟶	Increased number of legal wins for the environment
Unmet technology promises and false hope	⟵⟶	Aggressive net zero technology development and adoption
High carbon transport is the cheapest choice (for example, aviation or private cars)	⟵⟶	Low carbon transport is cheaper and more desirable
No investment in new builds and reliance on badly insulated old stock	⟵⟶	Investment in net zero compliant new builds and insulating old stock
Low occupancy and private ownership	⟵⟶	High occupancy sharing with communal ownership
Unregulated development planning and low investment in low carbon infrastructure and green spaces (low climate resilience)	⟵⟶	Strong development control and increasing investment in low carbon infrastructure and green spaces (high climate resilience)
Low fluctuating productivity and growth	⟵⟶	High steady productivity and growth
Increased urban-rural polarisation and high urban population pressures	⟵⟶	More equal population dispersal and less population pressures on urban centres
Flawed participatory mechanism resulting in no agreement on land use priorities	⟵⟶	Well-developed participatory mechanism to develop land use priorities
People feel that there is disproportionate impact (because of cost distribution of paying for net zero)	⟵⟶	People have trust and confidence that the cost of action is fairly distributed
National goals often in direct opposition to each other	⟵⟶	Common agreement between countries on 'priorities for humanity and the globe'
Society relies on manual tasks for day-to-day work	⟵⟶	Society relies on artificial intelligence and automated processes
Continuing decline in trust in politicians, government, and institutions	⟵⟶	Resurgence in trust in politicians, government, and institutions
Economic costs of climate change largely ignored in decision making	⟵⟶	Long- and short-term climate effects factored into decision making
Artificial intelligence plays an active role with higher risk of unintended consequences	⟵⟶	Artificial intelligence plays a primarily informative role but with coordinated oversight in active roles
Mercantilist attitude towards emissions	⟵⟶	Global commons attitude towards emissions
Economic centralisation	⟵⟶	Economic decentralisation and/or devolution
