# Supplementary material for: Policymaker-led scenarios and public dialogue facilitate energy demand analysis for net-zero futures
Source: Nat Energy. 2025 Nov 20;10(12):1482–92. doi: 10.1038/s41560-025-01898-3 (PMC12722179; doi:10.1038/s41560-025-01898-3)
Supplement: Supplementary file 2 — Reporting Summary [file 41560_2025_1898_MOESM2_ESM.pdf]

nature portfolio

Corresponding author(s): Maria Sharmina

Last updated by author(s): Sep 17, 2025

Reporting Summary

Nature Portfolio wishes to improve the reproducibility of the work that we publish. This form provides structure for consistency and transparency in reporting. For further information on Nature Portfolio policies, see our Editorial Policies and the Editorial Policy Checklist.

Statistics

For all statistical analyses, confirm that the following items are present in the figure legend, table legend, main text, or Methods section.

n/a	Confirmed	
☐	☒	The exact sample size (*n*) for each experimental group/condition, given as a discrete number and unit of measurement
☒	☐	A statement on whether measurements were taken from distinct samples or whether the same sample was measured repeatedly
☒	☐	The statistical test(s) used AND whether they are one- or two-sided<br>*Only common tests should be described solely by name; describe more complex techniques in the Methods section.*
☒	☐	A description of all covariates tested
☒	☐	A description of any assumptions or corrections, such as tests of normality and adjustment for multiple comparisons
☐	☒	A full description of the statistical parameters including central tendency (e.g. means) or other basic estimates (e.g. regression coefficient) AND variation (e.g. standard deviation) or associated estimates of uncertainty (e.g. confidence intervals)
☒	☐	For null hypothesis testing, the test statistic (e.g. *F*, *t*, *r*) with confidence intervals, effect sizes, degrees of freedom and *P* value noted<br>*Give P values as exact values whenever suitable.*
☒	☐	For Bayesian analysis, information on the choice of priors and Markov chain Monte Carlo settings
☒	☐	For hierarchical and complex designs, identification of the appropriate level for tests and full reporting of outcomes
☒	☐	Estimates of effect sizes (e.g. Cohen's *d*, Pearson's *r*), indicating how they were calculated

Our web collection on statistics for biologists contains articles on many of the points above.

Software and code

Policy information about availability of computer code

Data collection	All the data collection/data analysis software/tools used in the study are clearly mentioned in the manuscript. Data collection tools included: Mural, Excel, NVivo.
Data analysis	No new code or software was developed for this analysis. Sectoral and whole system models used to run each scenarios are described in the Methods section of this paper with appropriate referencing to extensive prior use. Data analysis tools included: TEAM-UK (Transport Energy Air pollution Model for the UK), UK National Housing Model (NHM), UK Building Energy Efficiency Survey (BEES) dataset, and UK Multi-Regional Input Output (UK-MRIO) model, Excel, NVivo.

For manuscripts utilizing custom algorithms or software that are central to the research but not yet described in published literature, software must be made available to editors and reviewers. We strongly encourage code deposition in a community repository (e.g. GitHub). See the Nature Portfolio guidelines for submitting code & software for further information.

Data

Policy information about availability of data

All manuscripts must include a data availability statement. This statement should provide the following information, where applicable:

- Accession codes, unique identifiers, or web links for publicly available datasets
- A description of any restrictions on data availability
- For clinical datasets or third party data, please ensure that the statement adheres to our policy

All results data underpinning the figures presented in the paper are available in the Supplementary Data 1 Excel sheet provided. Further graphs and analysis can be found at https://public.tableau.com/app/profile/stevepye#!/.https://public.tableau.com/app/profile/stevepye/vizzes#!/ . Demographic characteristics of public dialogue participants, including their numbers by gender, age, income, and geographic location, are available in Annex A of Patel et al. (2022).

Research involving human participants, their data, or biological material

Policy information about studies with human participants or human data. See also policy information about sex, gender (identity/presentation), and sexual orientation and race, ethnicity and racism.

Reporting on sex and gender	For expert workshops, no data was recorded on gender due to sampling of convenience (please see a justification in the 'Behavioural & social sciences study design' section). For public dialogue, the gender split between male and female was 44.1% and 55.9% respectively. The gender data for public dialogue participants was collected only to ensure a representative sample, and was not used in data analysis.
Reporting on race, ethnicity, or other socially relevant groupings	No race, ethnicity or other socially relevant data was used in the analysis.
Population characteristics	Please see the 'Behavioural & social sciences study design' section.
Recruitment	For expert workshops, participants were recruited by the policy project leads using an email-based snowballing approach reliant on existing network both internally to government departments and with external (academic, third sector) contacts. For public dialogue, participants were recruited through specialist recruitment agency partners: Criteria UK (an Approved IPSOS Supplier and regularly used for large deliberative projects across the UK) and Field Mouse (a specialist rural recruitment agency), using on-street, telephone, and online approaches, as well as snowballing.
Ethics oversight	The Government Office for Science (GOS) had ethics oversight for the expert workshops. Oversight for the public dialogue was shared between GOS and IPSOS, with the latter conducting the full process on behalf of the project team. A distinct element of our study compared to most academic-led research projects was an ongoing review by civil servants and by external experts, known to policy stakeholders as 'quality assurance'. The ongoing review included monthly submissions of drafts followed by presentations to the working group of junior civil servants, quarterly submissions of drafts followed by online presentations to a group of senior civil servants, and at least six-monthly online presentations to an external group of academic and industry experts ('expert advisory group', or EAG). While a core of external experts attended all EAG meetings, the composition of the EAG varied depending on whether the scrutiny was needed for the energy modelling or for the public dialogue. For the latter in particular, the EAG included experienced social scientists independent of the project team, helping to scrutinise the ethical and methodological aspects of public dialogue study design. The EAG members were also invited to attend the public dialogue workshops as observers. At the final stage of the project, a draft project report was reviewed by civil servants from a range of government departments to ensure consistency and relevance to policies outside the emissions and energy remit.

Note that full information on the approval of the study protocol must also be provided in the manuscript.

Field-specific reporting

Please select the one below that is the best fit for your research. If you are not sure, read the appropriate sections before making your selection.

☐ Life sciences ☒ Behavioural & social sciences ☐ Ecological, evolutionary & environmental sciences

For a reference copy of the document with all sections, see nature.com/documents/nr-reporting-summary-flat.pdf

Behavioural & social sciences study design

All studies must disclose on these points even when the disclosure is negative.

Study description	This was a mixed-method study involving expert workshops, public dialogue, and secondary data sources to parameterise sectoral simulation and whole systems optimisation models of the UK, including techno-economic data, energy balances, resource supply curves, and national statistics.
Research sample	Expert workshops included 35 stakeholders with relevant expertise from national government, local government, industry, third

Research sample	sector organisations, citizen groups and academia. This was a non-representative sample due to sampling of convenience. This sampling technique was chosen due to a relatively low number of experts (elite participants) and their limited availability to take part in the study. Public dialogue included 30 participants broadly representative of the UK population in gender, age, household income, location (urban or rural), housing type (owned or rented), degree of concern about climate change, attitude to technology adoption (e.g. early adopters), and attitude to government intervention.
Sampling strategy	We used sampling of convenience for expert workshops, and purposive sampling and minimum quotas for public dialogue. Both sampling strategies included snowballing (see the 'Recruitment' section above for more detail). Purposive sampling is often deemed to limit the generalisability of the findings as it can be difficult to ensure that the sample is representative. In our case, however, purposive sampling was specifically used with target quotes to make the sample representative of the UK population.
Data collection	A two-part facilitated online workshop with experts was held across two days, each part 2.5-hours long. A second three-hour facilitated online workshop was held with the same group of experts. The public dialogue data collection methodology included an introductory webinar, four three-hour facilitated workshops covering each scenario separately, and a final three-hour facilitated workshop bringing the scenarios together for comparison. All workshops took place online. Facilitators took notes during the workshops, and participants used an online platform (Mural) to jot down their ideas.
Timing	A two-part facilitated online workshop with experts was held across two days in February 2022, each part 2.5-hours long. A second three-hour facilitated online workshop was held in March 2022 with the same group of experts. The public dialogue was conducted in August-September 2022.
Data exclusions	No data was excluded from the analysis.
Non-participation	No participants dropped out from the study.
Randomization	Not applicable

Reporting for specific materials, systems and methods

We require information from authors about some types of materials, experimental systems and methods used in many studies. Here, indicate whether each material, system or method listed is relevant to your study. If you are not sure if a list item applies to your research, read the appropriate section before selecting a response.

Materials & experimental systems

n/a	Involved in the study
☒	☐ Antibodies
☒	☐ Eukaryotic cell lines
☒	☐ Palaeontology and archaeology
☒	☐ Animals and other organisms
☒	☐ Clinical data
☒	☐ Dual use research of concern
☒	☐ Plants

Methods

n/a	Involved in the study
☒	☐ ChIP-seq
☒	☐ Flow cytometry
☒	☐ MRI-based neuroimaging

Plants

Seed stocks	*Report on the source of all seed stocks or other plant material used. If applicable, state the seed stock centre and catalogue number. If plant specimens were collected from the field, describe the collection location, date and sampling procedures.*
Novel plant genotypes	*Describe the methods by which all novel plant genotypes were produced. This includes those generated by transgenic approaches, gene editing, chemical/radiation-based mutagenesis and hybridization. For transgenic lines, describe the transformation method, the number of independent lines analyzed and the generation upon which experiments were performed. For gene-edited lines, describe the editor used, the endogenous sequence targeted for editing, the targeting guide RNA sequence (if applicable) and how the editor was applied.*
Authentication	*Describe any authentication procedures for each seed stock used or novel genotype generated. Describe any experiments used to assess the effect of a mutation and, where applicable, how potential secondary effects (e.g. second site T-DNA insertions, mosiacism, off-target gene editing) were examined.*
